# Author Correction: Abiraterone acetate preferentially enriches for the gut commensal *Akkermansia muciniphila* in castrate-resistant prostate cancer patients

**DOI:** 10.1038/s41467-020-20410-x

**Published:** 2020-12-09

**Authors:** Brendan A. Daisley, Ryan M. Chanyi, Kamilah Abdur-Rashid, Kait F. Al, Shaeley Gibbons, John A. Chmiel, Hannah Wilcox, Gregor Reid, Amanda Anderson, Malcolm Dewar, Shiva M. Nair, Joseph Chin, Jeremy P. Burton

**Affiliations:** 1 Department of Microbiology and Immunology, The University of Western Ontario, London, ON N6A 5C1 Canada; 2 Canadian Centre for Human Microbiome and Probiotics Research, London, ON N6C 2R5 Canada; 3 Lawson Health Research Institute, St. Joseph’s Health Care London, London, ON N6A 4V2 Canada; 4 Department of Surgery, Division of Urology, Schulich School of Medicine, London, ON N6A 5C1 Canada

Correction to: *Nature Communications* 10.1038/s41467-020-18649-5, published online 24 September 2020.

This Article contained an error in the “Results” section. In the last sentence of the first paragraph of the “Results” section, the text indicated “Additional overview microbiota analyses identified Shannon’s diversity (a metric of bacterial community alpha diversity shown to be higher in PC patients compared to healthy controls^9^) to be significantly lower in ADT + AA samples compared to control samples (Supplementary Fig. 1A–C)”, which was an incorrect summary of the previous work in ref. ^9^ where alpha diversity was shown to be lower in PC patients compared to healthy control. The revised sentence reads:

“Additional overview analyses were performed on the basis of previously reported differences in GI microbiota alpha diversity between PC patients and healthy controls^9^. Shannon’s diversity (a metric of bacterial community alpha diversity accounting for species abundance and evenness) was found to be significantly lower in ADT + AA samples compared to control samples (Supplementary Fig. 1A–C).”

