# Supplementary material for: Abiraterone acetate preferentially enriches for the gut commensal Akkermansia muciniphila in castrate-resistant prostate cancer patients
Source: Nat Commun. 2020 Sep 24;11:4822. doi: 10.1038/s41467-020-18649-5 (PMC7515896; doi:10.1038/s41467-020-18649-5)
Supplement: Supplementary file 1 — Supplementary Information [file 41467_2020_18649_MOESM1_ESM.pdf]

Supplementary Information

Abirateronee acetate preferentially enriches for the gut commensal *Akkermansia muciniphila* in castrate-resistant prostate cancer patients

Daisley et al.

Supplementary Figures

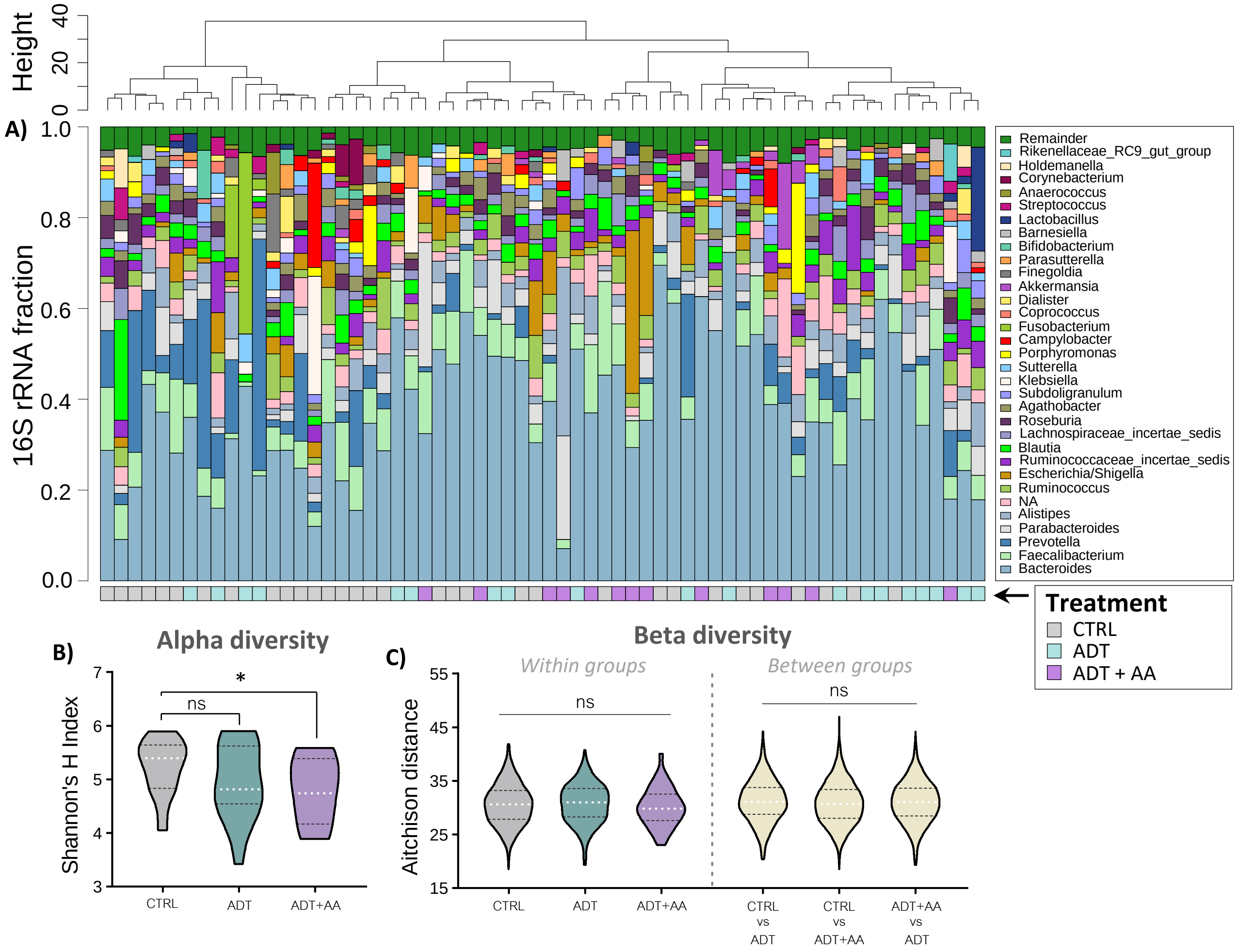

Supplementary Figure 1. Microbiota composition of prostate cancer patients receiving no treatment (CTRL), androgen-deprivation therapy alone (ADT), or androgen-deprivation therapy including abiraterone acetate (ADT+AA). (A) Bar plot of genus level microbiota composition for each individual. Hierarchal clustering of samples is shown in the dendrogram above the bar plot and was calculated using the "ward.D" method of the *hclust* function in R. Each bar represents a single patient sample. **(B)** Alpha diversity (measured via Shannon's H-Index) and **(C)** Beta diversity (measured via Aitchison's distance within and between treatment groups). Data shown as violin plots representing upper and low quartiles overlaid by kernel density estimations for n=68 patient samples. Statistical analysis shown for Kruskal-Wallis tests with multiple comparisons corrected using the Benjamini-Hochberg FDR method. *p=0.0401, ns = not significant.

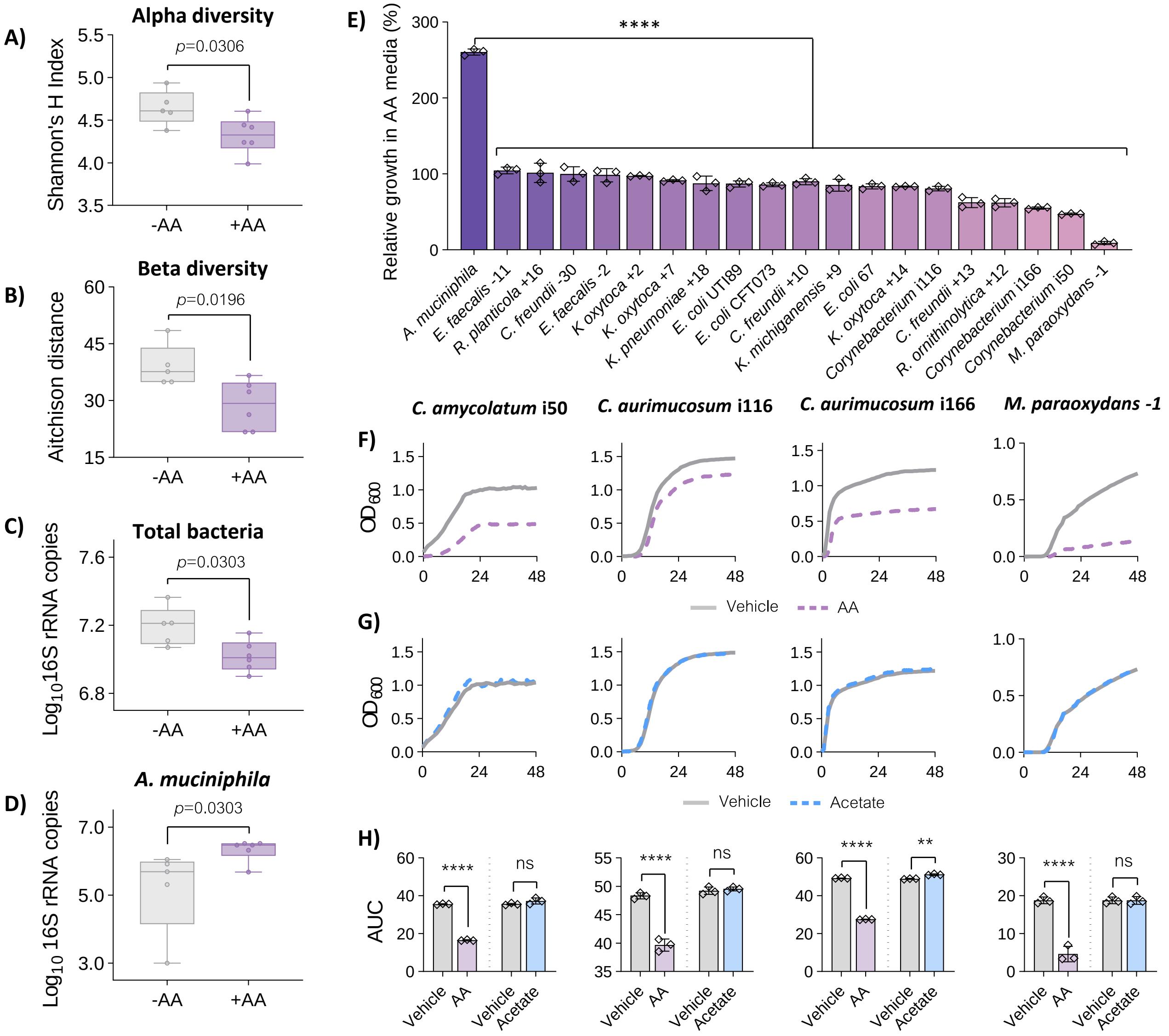

Supplementary Figure 2. Microbiota characteristics of the simulated gut model and *in vitro* growth assays for coryneform bacterial isolates. (A-B) Alpha diversity measured via Shannon's H-Index and **(B)** Beta diversity measured via Aitchison distance between samples within the same group. Statistical comparisons shown for two-tailed unpaired *t* tests with Welch's corrections. **(C)** Total bacteria and **(D)** *A. muciniphila* quantification by qPCR performed in technical duplicate using universal bacteria and species-specific primers, respectively. Statistical comparisons shown for two-tailed Wilcoxon-rank sum tests. All boxplot data represents the median (line in box), IQR (box), and minimum/maximum (whiskers) of n=5 non-AA exposure samples (-AA) and n=6 AA exposure samples (+AA). **(E)** Differences in bacterial growth were assessed at 24 h following incubation with 0.25 mM AA or vehicle. Data are expressed as percent growth (± standard deviation) and represent baseline-subtracted OD_{600} measurements in AA-supplemented media relative to vehicle controls. Statistics shown for one-way ANOVA with Tukey's multiple comparisons on n=3 biological replicates performed in technical triplicate. **(F-H)** Representative growth curves and area under curve (AUC) measurements for Actinobacteria isolates grown in media containing 0.25 mM AA or 0.25 acetate compared to respective vehicle controls. Data represents mean ± standard deviation of n=3 biological replicates performed in technical triplicate for each bacterial strain. Statistics shown for separate paired two-tailed *t* tests on n=3 biological replicates performed in technical triplicate. **p=0.0070, ****p<0.0001, ns= not significant.

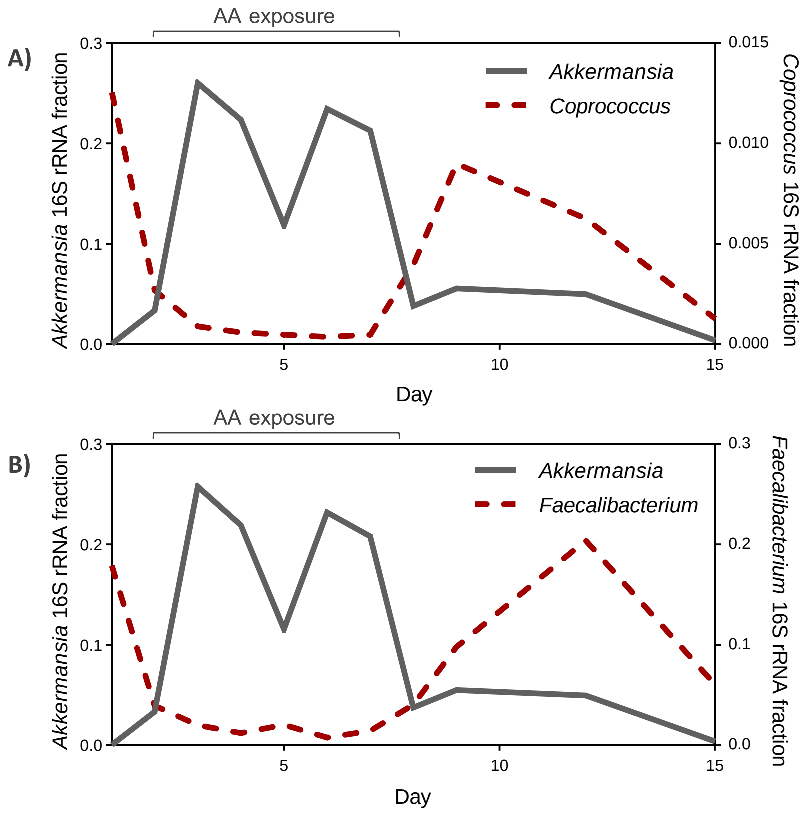

Supplementary Figure 3. Relative abundance of two major butyrate producers during AA exposure in the simulated gut model. Temporal overlay of relative abundance (shown as fraction of 16S rRNA copies) between *Akkermansia* and (A) *Coprococcus* or (B) *Faecalibacterium* during AA exposure in the simulated gut model.

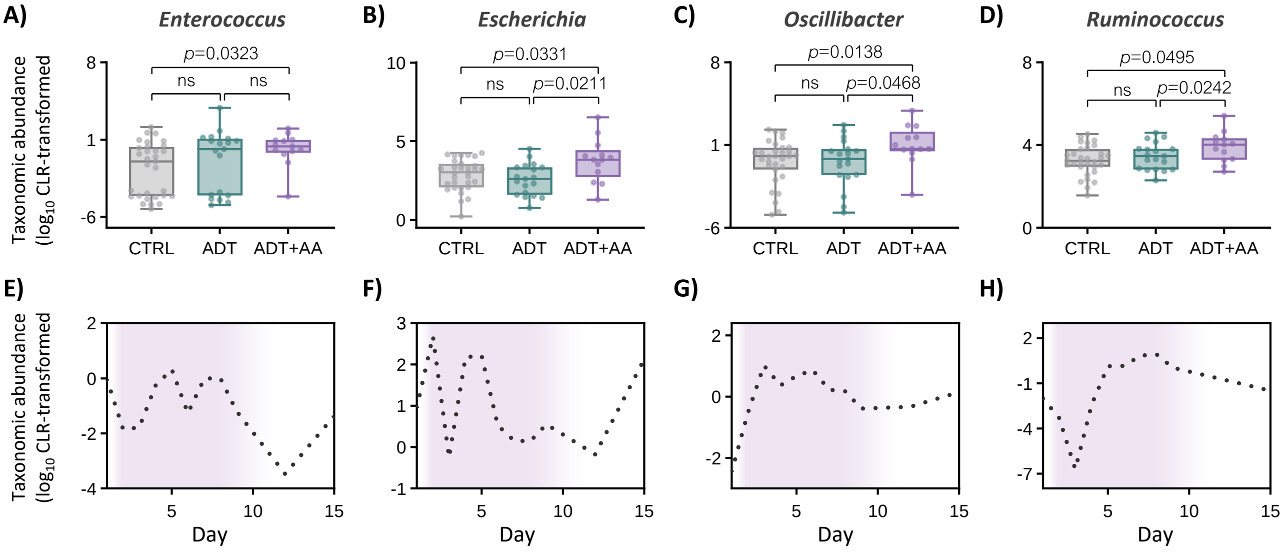

Supplementary Figure 4. Similarities in taxonomic abundance between prostate cancer patients and the simulated gut model. Relative abundance of bacteria in the microbiota of **(A-D)** AA-treated patients. Data represents the median (line in box), IQR (box), and minimum/maximum (whiskers) of n=68 prostate cancer patient samples. Statistical comparisons shown for two-tailed Wilcoxon-rank sum tests with multiple comparisons corrected using ALDEx2 differential relative abundance analysis software. **(E-H)** AA-exposed gut model samples. Purple sections indicate the AA exposure period. ns = not significant.

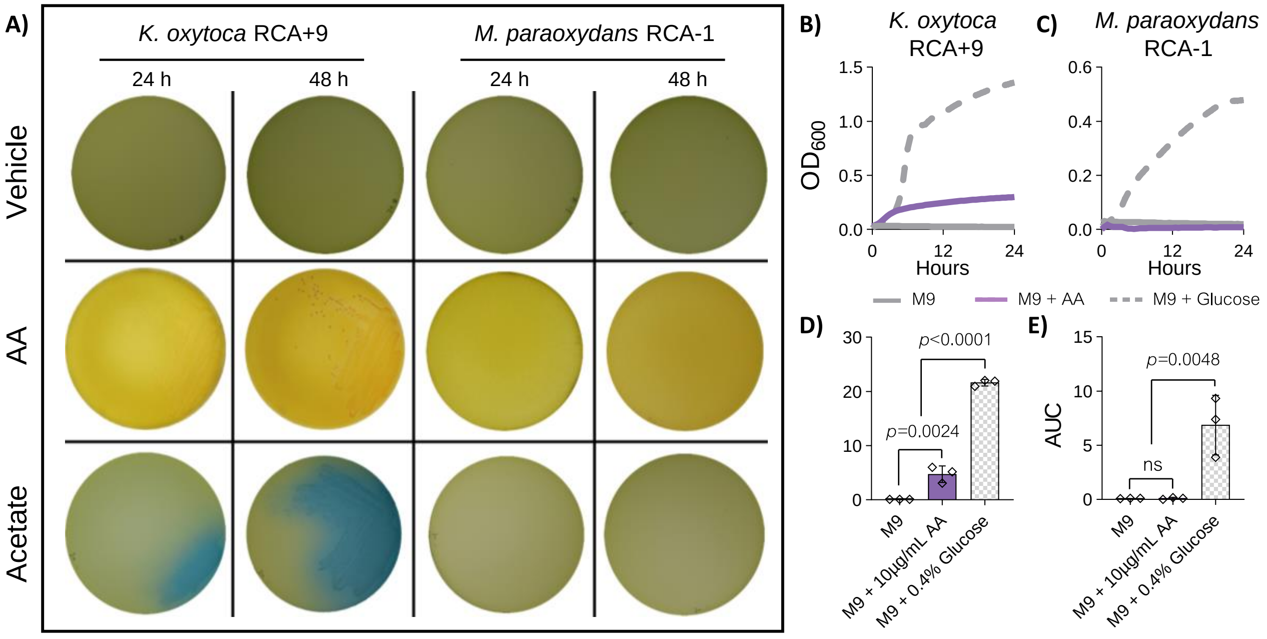

Supplementary Figure 5. Screening of bacterial isolates for the ability to use AA or acetate as a sole carbon source. (A) Bacterial isolates were grown on colorimetric agar plates with AA, acetate, or vehicle. Growth was subsequently monitored with representative photographs shown from 24 and 48 h following incubation. Yellow (AA plates) and blue (acetate plates) color change indicates the utilization of acetate. **(B-C)** Growth curves and **(D-E)** area under the curve (AUC) measurement of bacterial isolates grown in M9 minimal salts media supplemented with vehicle and either 10 μg/mL AA or 0.4% glucose as a positive control. Data represents mean ± standard deviation (one-way ANOVA with Tukey's multiple comparisons) of n=3 biological replicates performed in technical triplicate for each bacterial strain. ns= not significant.

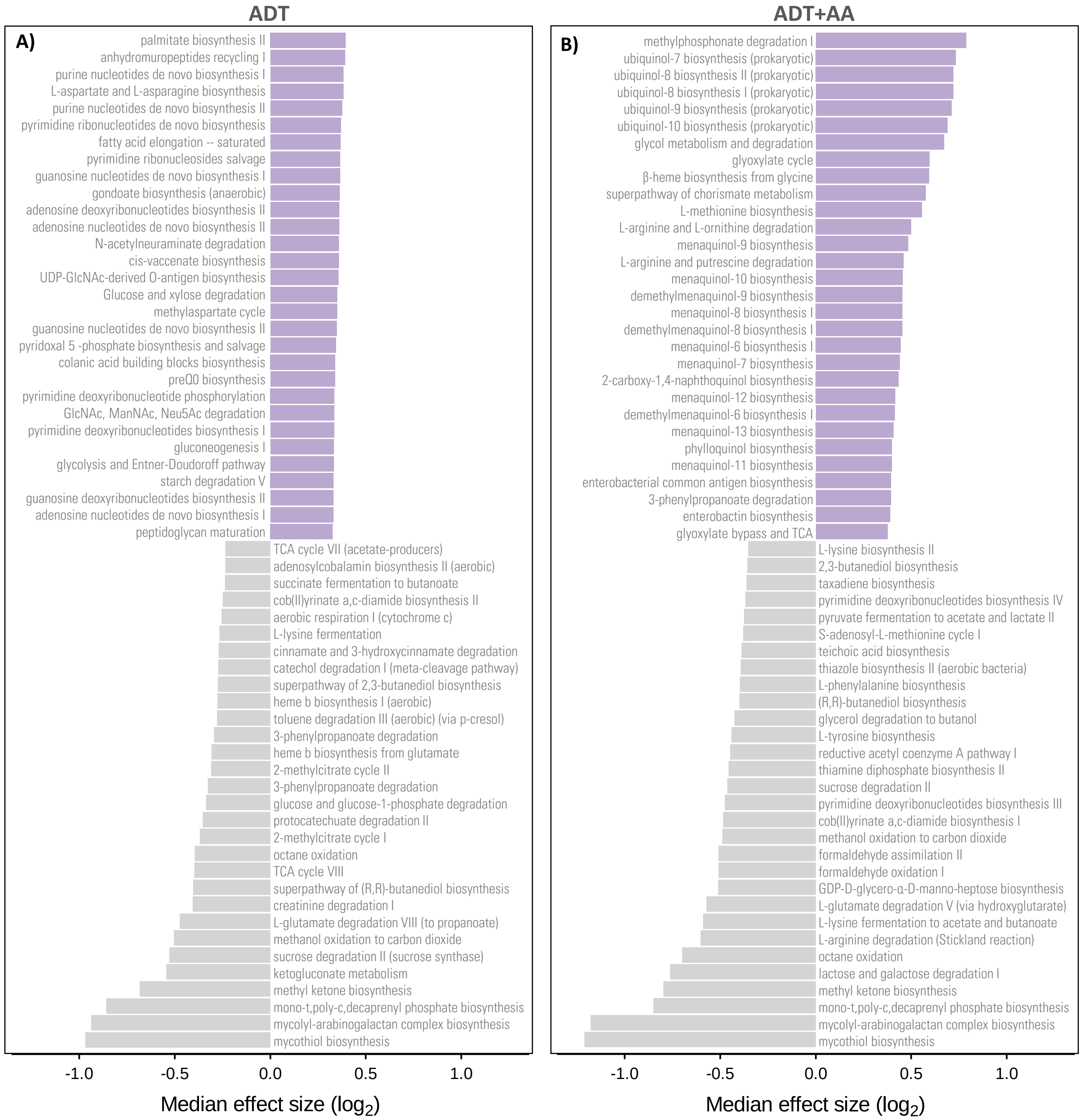

Supplementary Figure 6. Predicted bacterial metagenome pathway abundances from prostate cancer patient rectal swab samples. Effect size plots for **(A)** ADT and **(B)** ADT+AA groups are relative to no treatment control patients. Predicted pathways were inferenced using an exact sequence variant approach in *PICRUSt2* software and then annotated using the Metacyc metabolic pathway database. Additive boosted general linear models were used to calculate significance with *MaAsLin2* software and effect size was determined using *ALDEx2* software.

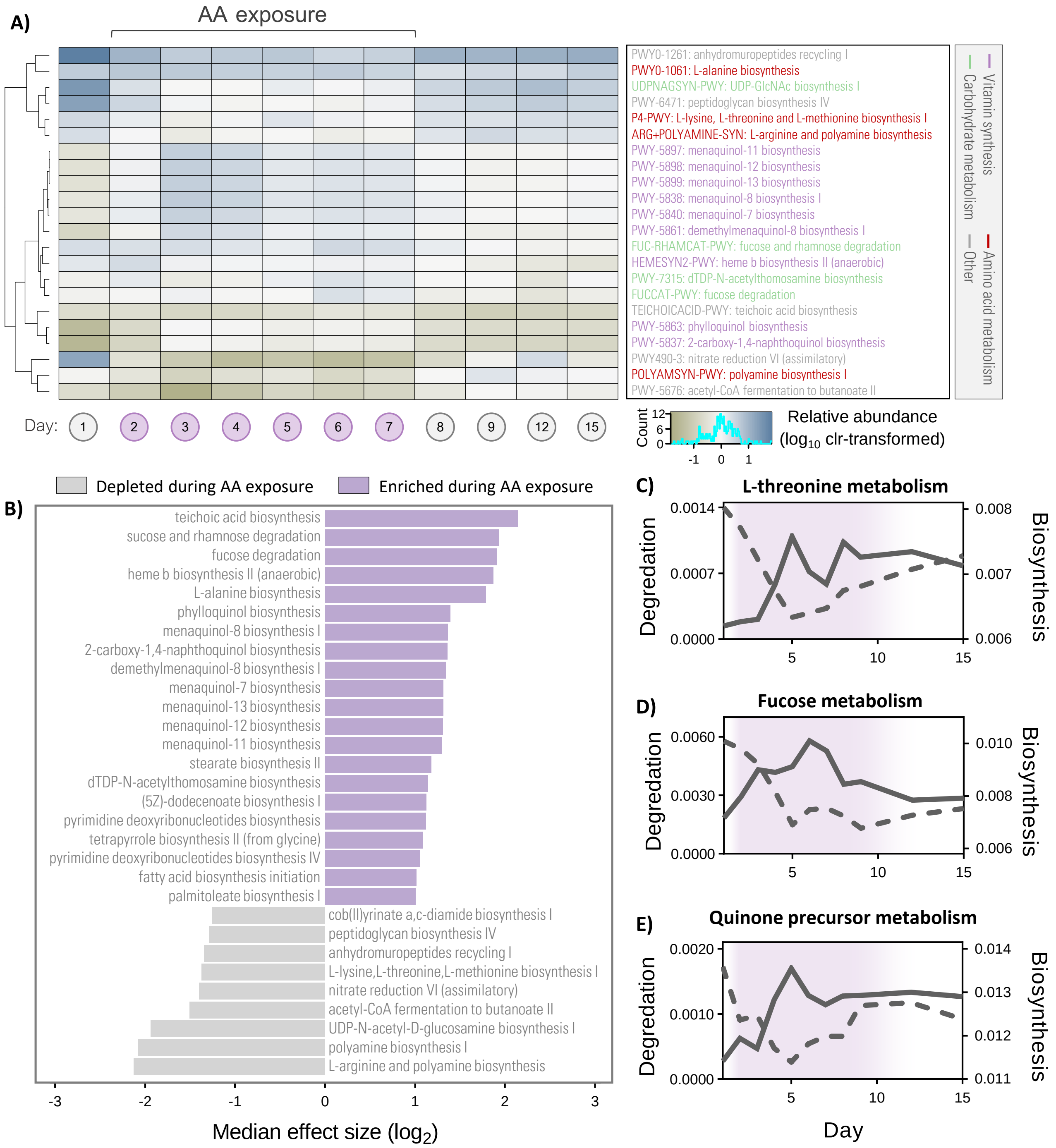

Supplementary Figure 7. Predicted bacterial metagenome in a simulated distal gut model during AA exposure. (A-B) Heatmap and effect plot of top predicted pathways determined to be differentially abundant in the simulated gut model during AA exposure. Hierarchal clustering of samples is shown in the dendrogram beside the heatmap and was calculated using the "complete" method of the *hclust* function in R. **(C-E)** Temporal overlay graphs showing relevant degradation and biosynthesis pathway abundances in gut model samples over time. Purple sections indicate the AA exposure period. Predicted pathways were inferenced using an exact sequence variant approach with *PICRUSt2* software and annotated using the Metacyc metabolic pathway database. Statistical comparisons were performed using a Welch's *t*-test (with multiple comparisons corrected by Benjamini-Hochberg FDR) and effect size was determined using *ALDEx2* software. Solid lines = degradation-related pathways, Dotted lines = biosynthesis-related pathways.

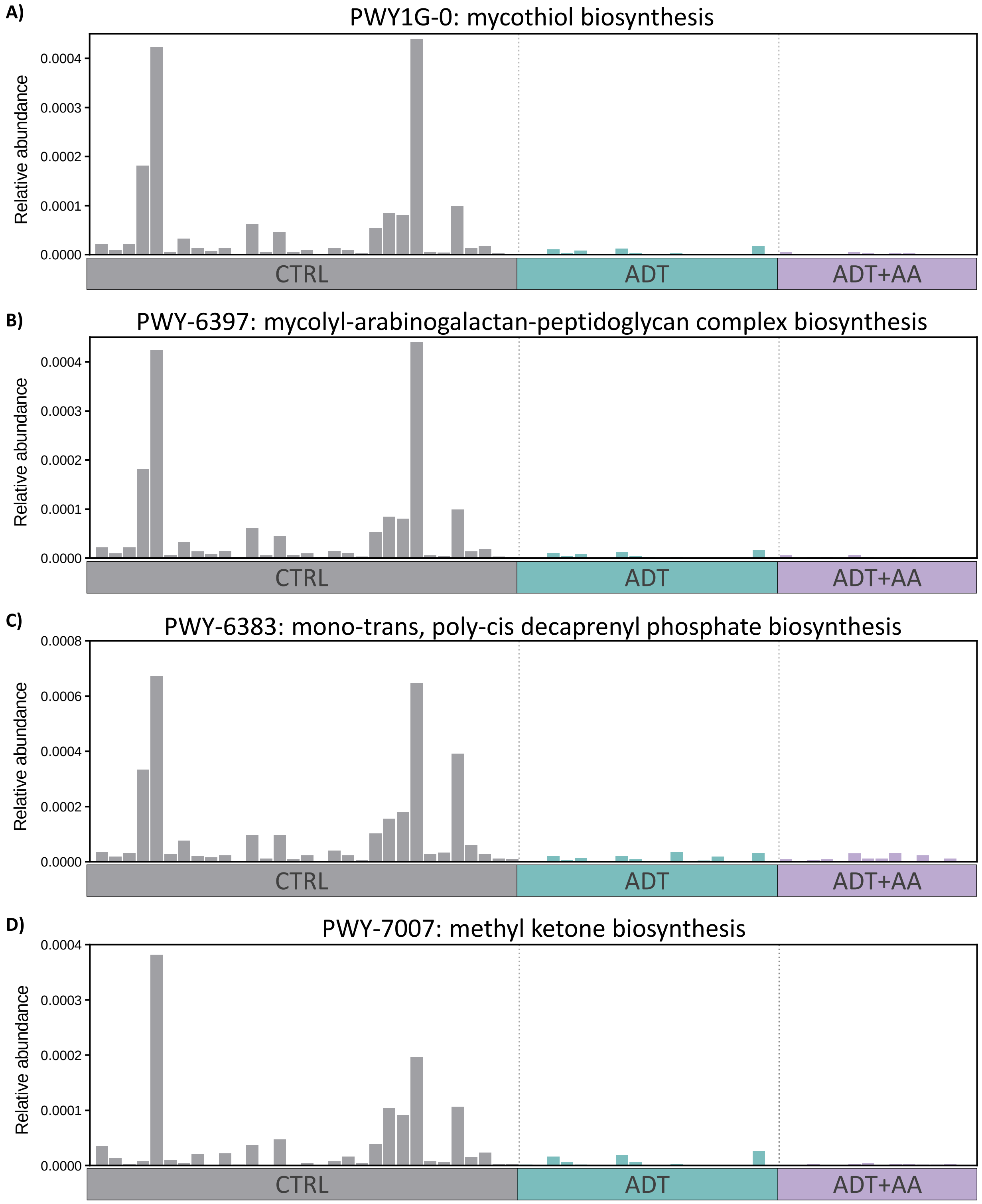

Supplementary Figure 8. Host androgen suppression depletes metabolic pathways related to mycolic acid biosynthesis in the gut of prostate cancer patients. Relative pathway abundance in comparison to 306 total pathways evaluated. Bacterial metabolic pathways were inferenced using an exact sequence variant approach with *PICRUSt2* software and were annotated using the Metacyc pathway database. CTRL = no treatment (n=33), ADT = androgen deprivation therapy (n=21), ADT+AA = ADT with abiraterone acetate (n=14).

Supplementary Tables

Supplementary Table 1. Demographic and clinical variables of prostate cancer patients in this study.

**Variable**	**No treatment controls (CTRL)**	**Androgen deprivation therapy (ADT)**	**ADT + abiraterone acetate (ADT+AA)**
Number of participants	33	21	14
Mean age ± SD (range)	70.07±7.76 (54-83)	75.86±9.47 (56-89)	73.45±10.64 (55-92)
Mean height ± SD (range)	1.74±0.07m (1.65-1.91)	1.72±0.09m (1.53-1.88)	1.74±0.08m (1.55-1.86)
Mean weight ± SD (range)	84.49±14.27kg (59.60-112.40)	86.37±15.98kg (59.90-123.40)	88.85±15.50kg (66.00-124.80)
Mean BMI ± SD (range)	27.85±3.75 (21.63-35.03)	29.16±5.70 (21.32-40.29)	28.30±3.30 (21.31-31.44)
Inflammatory bowel disease	2	1	1
Pelvic radiation exposure	15	13	11
Metastatic disease	0	12	14
Medications			
Prednisone	0	4	13
Dexamethasone	0	1	1
Metformin	4	3	4
Bicalutamide	0	5	1
Antibiotics (<3 months)	7	3	0
Primary Treatment			
Active surveillance	5	0	0
Radical prostatectomy	20	13	8
Radiation	7	5	2
De novo mets	0	4	4
TULSA*	1	0	0

Notes: One patient in the ADT group was receiving two primary treatments. *Transurethral ultrasound ablation

Supplementary Table 2. Effect of clinical metadata variables on patient microbiota variation.

**Variable**	**PC1**	**PC2**	**R^2**	**Pr(>r)**
Age	0.7337	-0.6795	0.0368	0.4100
Androgen deprivation therapy (ADT)	-0.9997	-0.0230	0.1188	**0.0430***
Abiraterone acetate (AA) treatment	0.9996	-0.0298	0.2247	**0.0010*****
Height	0.8268	-0.5626	0.0049	0.8900
Weight	-0.9948	0.1014	0.0295	0.4720
BMI	-0.9785	0.2063	0.0441	0.3310
Corticosteroids	0.9009	-0.4341	0.1158	**0.0490***
Antibiotics (<3 months)	-0.9422	-0.3352	0.1042	0.0560·
Bicalutamide (<3 months)	-0.8156	-0.5786	0.0305	0.5160
Metformin	-0.5511	0.8344	0.0033	0.9200
Inflammatory bowel disease	-0.8344	-0.5512	0.0006	0.9820
Pelvic radiation	0.3497	0.9369	0.0525	0.2830
Primary treatment	0.5418	-0.8405	0.0429	0.3670
Disease status	0.8775	-0.4795	0.0397	0.3590
PSA levels	-0.8999	0.4362	0.0128	0.8130
Metastatic disease	0.8798	0.4754	0.0508	0.3030

Supplementary Table 3. MaAsLin2 genus-level associations with AA exposure in the simulated gut model.

Variable	Genus	coef	stderr	N	N.not.0	pval[a]	qval[b]
abi_exposure	Akkermansia	3.523258	1.061189	11	9	0.010538439	0.038434308
abi_exposure	Hungatella	2.111551	0.613834	11	7	0.00882457	0.03419521
hour	Lachnospiraceae;NA	2.00053	0.344927	11	2	0.000405227	0.006953002
hour	Ruminiclostridium	1.96439	0.41751	11	3	0.001531516	0.013564853
hour	Ruminococcaceae_UCG-005	1.795329	0.432573	11	2	0.003207444	0.019886152
hour	Christensenellaceae_R-7_group	1.614126	0.285861	11	3	0.00048336	0.006953002
hour	Negativicoccus	1.477502	0.375322	11	2	0.004316295	0.024328206
abi_exposure	Klebsiella	1.474786	0.389314	11	11	0.005325772	0.025486191
abi_exposure	Oscillibacter	1.419951	0.484826	11	7	0.019033602	0.059004165
hour	UBA1819	1.285591	0.494791	11	3	0.031703833	0.084062534
hour	Ruminococcus	1.196444	0.600136	11	5	0.081315562	0.173847063
abi_exposure	Bilophila	0.846775	0.223672	11	7	0.005343879	0.025486191
hour	Intestinimonas	0.72566	0.238562	11	5	0.016019083	0.052272796
abi_exposure	Dialister	0.589792	0.346461	11	11	0.127103307	0.225154429
hour	Stenotrophomonas	0.553773	0.214516	11	3	0.032540336	0.084062534
hour	Oscillibacter	0.407065	0.226462	11	7	0.109972486	0.206614973
hour	Klebsiella	0.333515	0.181849	11	11	0.103993577	0.201487556
hour	Lachnoclostridium	0.198725	0.103529	11	11	0.091184561	0.182369122
hour	Blautia	-0.46135	0.236391	11	11	0.086770528	0.179325759
hour	Faecalibacterium	-0.47163	0.220658	11	11	0.065040093	0.144017348
hour	Subdoligranulum	-0.58577	0.106128	11	10	0.000560726	0.006953002
hour	Coprococcus	-0.59666	0.34557	11	2	0.122512794	0.223405683
hour	Parabacteroides	-0.6257	0.178937	11	11	0.008117321	0.033551594
abi_exposure	Clostridium_sensu_stricto_1	-0.6412	0.392175	11	6	0.140691197	0.242301506
hour	Bacteroides	-0.67432	0.15805	11	11	0.002737186	0.019886152
hour	Clostridium_sensu_stricto_1	-0.67496	0.183185	11	6	0.006178028	0.027359836
abi_exposure	Citrobacter	-0.94505	0.330515	11	11	0.021173153	0.061243914
abi_exposure	Stenotrophomonas	-1.0609	0.459251	11	3	0.049683483	0.118475997
hour	Bilophila	-1.27291	0.104477	11	7	1.91E-06	5.92E-05
hour	Alistipes	-1.55086	0.109649	11	4	6.07E-07	3.76E-05
abi_exposure	Coprococcus	-1.68031	0.739822	11	2	0.052788489	0.121218012
hour	Agathobacter	-1.84333	0.359054	11	4	0.000891832	0.009215601
abi_exposure	Faecalibacterium	-1.9865	0.472401	11	11	0.002975645	0.019886152
abi_exposure	Ruminiclostridium	-2.09394	0.893836	11	3	0.047220704	0.117107345
abi_exposure	Lachnospiraceae;NA	-2.09895	0.738445	11	2	0.021731711	0.061243914
hour	Fusicatenibacter	-2.41615	0.792162	11	3	0.015819257	0.052272796

[a]MaAsLin2 GLM, [b]MaAsLin2 GLM BH-corrected

Supplementary Table 4. Bacterial isolates showing a capacity to utilize AA or acetate as a sole carbon source.

Designation	No carbon source	Abiraterone acetate	Acetate only	Top bacterial candidate	Percent identity	Percent coverage
RCA+1	-	+	+	*Citrobacter freundii*	97%	92%
RCA+2/PCp7	-	+	+	*Klebsiella oxytoca*	93%	91%
RCA+3	-	+	+	*Citrobacter freundii*	96%	87%
RCA+4	-	+	+	*Klebsiella oxytoca*	98%	91%
RCA+5	-	+	+	*Citrobacter freundii*	96%	100%
RCA+6	-	+	+	*Citrobacter freundii*	97%	87%
RCA+7/PCp8	-	+	+	*Klebsiella oxytoca*	99%	92%
RCA+8	-	+	+	*Klebsiella oxytoca*	99%	87%
RCA+9/PCp9	-	+	+	*Klebsiella michiganensis*	93%	94%
RCA+10/PCp1	-	+	+	*Citrobacter freundii*	96%	90%
RCA+11	-	+	+	*Klebsiella michiganensis*	93%	89%
RCA+12	-	+	+	*Raoultella ornithinolytica*	97%	100%
RCA+13/PCp2	-	+	+	*Citrobacter freundii*	97%	88%
RCA+14/PCp10	-	+	+	*Klebsiella oxytoca*	99%	92%
RCA+15	-	+	+	*Citrobacter freundii*	98%	87%
RCA+16	-	+	+	*Raoultella planticola*	98%	94%
RCA+17	-	+	+	*Raoultella ornithinolytica*	97%	97%
RCA+18/PCp11	-	+	+	*Klebsiella pneumoniae*	98%	93%
RCA+19	-	+	+	*Citrobacter freundii*	96%	88%
RCA+20/PCp4	-	+	+	*Klebsiella oxytoca*	99%	90%
RCA+21/PCp5	-	+	+	*Raoultella planticola*	98%	90%
RCA+22/PCp6	-	+	+	*Raoultella ornithinolytica*	98%	95%
RCA+23	-	+	+	*Citrobacter freundii*	97%	94%
RCA+24	-	+	+	*Klebsiella oxytoca*	90%	84%
RCA-1	-	-	-	*Microbacterium paraoxydans*	63%	50%
RCA-3	-	-	-	*Enterococcus gilvus*	98%	90%
RCA-6	-	-	-	*Microbacterium resistens*	97%	94%
RCA-7	-	-	-	*Enteroccocus avium*	98%	89%
RCA-11	-	-	-	*Enterococcus faecalis*	98%	94%
RCA-30/PCp3	-	-	-	*Citrobacter freundii*	86%	85%

Notes: (+) indicates growth, (-) indicates no growth

Supplementary Table 5. LEfSe multiclass analysis of bacterial metabolic pathway abundance in prostate cancer patient samples.

MetaCyc Pathway	Log10 highest class average	Class	*p*-value[a]	Effect size[b]
CALVIN-PWY	3.939021848	-AA	0.000107774	-2.508527287
PWY-5100	3.883085167	-AA	0.000243904	-2.521224538
PWY-5345	3.394707539	+AA	0.000926612	2.630778291
PWY-5857	2.855794053	+AA	0.001110365	2.310533824
PWY-5855	2.855794053	+AA	0.001110365	2.310533824
PWY-6708	2.855794053	+AA	0.001110365	2.310533824
PWY-5856	2.855794053	+AA	0.001110365	2.310533824
UBISYN-PWY	2.853945506	+AA	0.001178746	2.388108699
PWY0-1533	2.761915563	+AA	0.002645388	2.264341186
PWY-6385	3.868950215	-AA	0.005632927	-2.337569741
PWY-6386	3.877825894	-AA	0.005632927	-2.361226484
PWY-6387	3.876436673	-AA	0.007285672	-2.324983018
PEPTIDOGLYCANSYN-PWY	3.87182306	-AA	0.008060423	-2.325949167
COA-PWY	3.848245392	-AA	0.008474802	-2.265150367
GLYCOL-GLYOXDEG-PWY	2.70527481	+AA	0.009361138	2.169541226
PWY-6609	3.869450139	-AA	0.009361138	-2.410185506
TRNA-CHARGING-PWY	3.873548612	-AA	0.012536526	-2.258136775
PWY-6123	3.885290309	-AA	0.012536526	-2.283646552
GLYOXYLATE-BYPASS	2.882696652	+AA	0.013149787	2.382655939
PWY-5686	3.913954247	-AA	0.015151634	-2.251715708
PWY-5838	3.06528126	+AA	0.01741713	2.396553442
PWY-5840	3.061612717	+AA	0.01741713	2.319616216
PWY-5897	3.031339829	+AA	0.01741713	2.314028067
PWY-5898	3.031339829	+AA	0.01741713	2.314028067
PWY-5899	3.031339829	+AA	0.01741713	2.314028067
PWY-5861	2.954107734	+AA	0.019974352	2.25020226
SO4ASSIM-PWY	3.300842179	+AA	0.021856009	2.33660941
PWY-6122	3.895735936	-AA	0.021856009	-2.221182232
PWY-6277	3.895735936	-AA	0.021856009	-2.221182232
PWY-5837	2.704710279	+AA	0.023890061	2.320587025
PWY-5863	2.754032548	+AA	0.023890061	2.315166462
PWY-5920	2.772679252	+AA	0.023890061	2.26937206
SULFATE-CYS-PWY	3.517949975	+AA	0.028454972	2.42699335
PWY-6126	3.925157916	-AA	0.029707245	-2.273962614
PWY-7229	3.94157387	-AA	0.031006619	-2.305394309
ALL-CHORISMATE-PWY	2.810055828	+AA	0.033752223	2.179354054
PWY-6151	3.764083207	-AA	0.036703121	-2.5019987
PWY-6121	3.908382685	-AA	0.038259196	-2.17481055
PWY-5845	2.833231382	+AA	0.043267913	2.202026885
PWY-7208	3.924010134	-AA	0.046906198	-2.246176243

Notes: (-AA) indicate not receiving abiraterone acetate, (+AA) indicates receiving abiraterone acetate
[a] LEfSe linear discriminant analysis
[b] LEfSe effect size

Supplementary Table 6. Primers used for qPCR-based quantification of bacterial loads in prostate patient rectal swab and chemostat samples.

Target	NCBI ID	Forward sequence (5'-3')	Reverse sequence (5'-3')	Ref
Universal bacteria	TXID_2	GTATTACCGCGGCTGCTGG	ACTCCTACGGGAGGCAGCAG	[1]
A. municiphilia	TXID_239935	CAGCACGTGAAGGTGGGGAC	CCTTGCGGTTGGCTTCAGAT	[2]
Enterobacteriaceae	TXID_543	ATGTTACAACCAAAGCGTACA	TTACCYTGACGCTTAACTGC	[3]

Supplementary References

1. Yu, H. *et al.* Urinary microbiota in patients with prostate cancer and benign prostatic hyperplasia. *Arch. Med. Sci.* **11**, 385–394 (2015).
2. Roopchand, D. E. *et al.* Dietary polyphenols promote growth of the gut bacterium *Akkermansia muciniphila* and attenuate high-fat diet–induced metabolic syndrome. *Diabetes* **64**, 2847–2858 (2015).
3. Takahashi, H. *et al.* Development of quantitative real-time PCR for detection and enumeration of Enterobacteriaceae. *Int. J. Food Microbiol.* **246**, 92–97 (2017).
