# Supplementary material for: Abiraterone acetate preferentially enriches for the gut commensal Akkermansia muciniphila in castrate-resistant prostate cancer patients
Source: Nat Commun. 2020 Sep 24;11:4822. doi: 10.1038/s41467-020-18649-5 (PMC7515896; doi:10.1038/s41467-020-18649-5)
Supplement: Supplementary file 3 — Descriptions of Additional Supplementary Files [file 41467_2020_18649_MOESM3_ESM.pdf]

Descriptions of Additional Supplementary Files

Supplementary Data 1

Description: MaAsLin2 genus-level associations with significant clinical metadata variables in PC patient microbiota.

Supplementary Data 2

Description: ALDEx2 effect sizes for pathway abundances of predicted bacterial metagenomes in ADT relative to CTRL patient rectal swab samples.

Supplementary Data 3

Description: ALDEx2 effect sizes for pathway abundances of predicted bacterial metagenomes in ADT+AA relative to CTRL patient rectal swab samples.

Supplementary Data 4

Description: MaAsLin2 associations between prostate cancer treatment group and pathway abundance of predicted bacterial metagenomes in patient rectal swab samples.

Supplementary Data 5

Description: ALDEx2 effect sizes for predicted metabolic pathways in AA-exposed gut model samples.

Supplementary Data 6

Description: MaAsLin2 associations between AA exposure and metabolic pathway abundances in simulated gut model samples.

Supplementary Data 7

Description: Read count table stratified by taxonomy for PICRUSt2 predicted bacterial metagenome of PC patient samples.

Supplementary Data 8

Description: Read count table stratified by taxonomy for PICRUSt2 predicted bacterial metagenome of simulated gut samples.
