# Supplementary material for: Abiraterone acetate preferentially enriches for the gut commensal Akkermansia muciniphila in castrate-resistant prostate cancer patients
Source: Nat Commun. 2020 Sep 24;11:4822. doi: 10.1038/s41467-020-18649-5 (PMC7515896; doi:10.1038/s41467-020-18649-5)
Supplement: Supplementary file 12 — Reporting Summary [file 41467_2020_18649_MOESM12_ESM.pdf]

nature research

Corresponding author(s): Jeremy Burton

Last updated by author(s): Aug 22, 2020

Reporting Summary

Nature Research wishes to improve the reproducibility of the work that we publish. This form provides structure for consistency and transparency in reporting. For further information on Nature Research policies, see our Editorial Policies and the Editorial Policy Checklist.

Statistics

For all statistical analyses, confirm that the following items are present in the figure legend, table legend, main text, or Methods section.

n/a	Confirmed	
☐	☒	The exact sample size (*n*) for each experimental group/condition, given as a discrete number and unit of measurement
☐	☒	A statement on whether measurements were taken from distinct samples or whether the same sample was measured repeatedly
☐	☒	The statistical test(s) used AND whether they are one- or two-sided<br>*Only common tests should be described solely by name; describe more complex techniques in the Methods section.*
☐	☒	A description of all covariates tested
☐	☒	A description of any assumptions or corrections, such as tests of normality and adjustment for multiple comparisons
☐	☒	A full description of the statistical parameters including central tendency (e.g. means) or other basic estimates (e.g. regression coefficient) AND variation (e.g. standard deviation) or associated estimates of uncertainty (e.g. confidence intervals)
☐	☒	For null hypothesis testing, the test statistic (e.g. *F*, *t*, *r*) with confidence intervals, effect sizes, degrees of freedom and *P* value noted<br>*Give P values as exact values whenever suitable.*
☐	☒	For Bayesian analysis, information on the choice of priors and Markov chain Monte Carlo settings
☐	☒	For hierarchical and complex designs, identification of the appropriate level for tests and full reporting of outcomes
☐	☒	Estimates of effect sizes (e.g. Cohen's *d*, Pearson's *r*), indicating how they were calculated

Our web collection on statistics for biologists contains articles on many of the points above.

Software and code

Policy information about availability of computer code

Data collection	Quant Studio 5 Real-Time PCR System (Applied Biosystems) software was used to collect qPCR data. MiSeq (Illumina) software was used to collect 16S rRNA sequencing data.
Data analysis	GraphPad Prism (v8.3) was used for statistical analyses throughout the manuscript with each of the statistical measures described in the methods section and figure legends where appropriate. ChemStation (vA.10.02) was used for HPLC analysis of abiraterone breakdown assays. QuantStudio Design and Analysis v 1.4 software (Applied Biosytems) was used to analyze qPCR data. DADA2 (v1.8), ALDEx2 (v1.18), PICRUSt (v2.0), Cytoscape (v3.7.2), MaAsLin2 (v0.99.1), vegan (v2.5-6), QIIME2 (v2020.2), zCompositions (v1.3.2-1), CoDaSeq (v0.99.4), LEfSe (v1.0), growthcurver (v0.3.0), decontam (v.1.1.2), and R (v3.6.0) softwares were used to analyze and/or visualize 16S rRNA sequencing data in accordance with standard settings using the specific functions described in the Methods section of the manuscript.

For manuscripts utilizing custom algorithms or software that are central to the research but not yet described in published literature, software must be made available to editors and reviewers. We strongly encourage code deposition in a community repository (e.g. GitHub). See the Nature Research guidelines for submitting code & software for further information.

Data

Policy information about availability of data

All manuscripts must include a data availability statement. This statement should provide the following information, where applicable:

- Accession codes, unique identifiers, or web links for publicly available datasets
- A list of figures that have associated raw data
- A description of any restrictions on data availability

Raw sequence reads were uploaded to the NCBI Sequence Read Archive and are accessible under BioProject ID PRJNA609050 [https://www.ncbi.nlm.nih.gov/bioproject/PRJNA609050]. Figures 1-4 and Supplementary Figures 1-4,6-8 are associated with this raw data. Source data underlying these figures has also been

made available in Supplementary Data 1, Supplementary Tables 3&5, and Supplementary Data 2-8. All other remaining relevant data are provided in the article, supplementary information, or available from the corresponding author upon reasonable request.

Field-specific reporting

Please select the one below that is the best fit for your research. If you are not sure, read the appropriate sections before making your selection.

- [x] Life sciences
- [ ] Behavioural & social sciences
- [ ] Ecological, evolutionary & environmental sciences

For a reference copy of the document with all sections, see nature.com/documents/nr-reporting-summary-flat.pdf

Life sciences study design

All studies must disclose on these points even when the disclosure is negative.

Sample size	Sample size was restricted based on enrollment in the Canadian Observational Study in Metastatic Cancer of the Prostate (COSMiC; NCT02364531). The 68 patients examined in this study were the only patients from the COSMIC cohort that were successfully recruited for purposes of microbiota analysis. This study is the largest of its kind and the sample size is over double that of previous investigations on the microbiota of prostate cancer patients.
Data exclusions	Standard quality assurance measures were performed on the 16S rRNA gene sequencing dataset in the DADA2 which resulted in removal of poor quality reads and chimeras from sample sequence reads. Outliers were identified within groups using the "codaSeq.outlier" function in the CoDaSeq R package. We have disclosed this in the methods sections and ensured that all raw sequencing material uploaded to the NCBI Sequence Read Archive include these exclusions.
Replication	The clinical study findings were replicated in a host-free gut model [a single run with n=11 time points across 15 days after an initial stabilization period of 14 days). In addition, a set of n=8 fecal samples collected from patients were incubated with media supplemented with or without abiraterone acetate followed by 16S rRNA sequencing to validate AA effects on human-derived microbial communities with high or low levels of background Akkermansia. As outlined in the manuscript, results from in vivo and in vitro were highly consistent with one another in regards to how AA impacted overall microbiota composition.
Randomization	The COSMiC study is a non-interventional, multicenter prospective, observational study in which patients may receive diagnostic, therapeutic, or other types of interventions, but the investigator does not assign participants to specific interventions.
Blinding	Not applicable as investigators did not assign participants to groups. Sample collection and statistical analyses were performed by different individuals.

Reporting for specific materials, systems and methods

We require information from authors about some types of materials, experimental systems and methods used in many studies. Here, indicate whether each material, system or method listed is relevant to your study. If you are not sure if a list item applies to your research, read the appropriate section before selecting a response.

Materials & experimental systems

n/a	Involved in the study	
x		Antibodies
x		Eukaryotic cell lines
x		Palaeontology and archaeology
x		Animals and other organisms
	x	Human research participants
	x	Clinical data
x		Dual use research of concern

Methods

n/a	Involved in the study	
x		ChIP-seq
x		Flow cytometry
x		MRI-based neuroimaging

Human research participants

Policy information about studies involving human research participants

Population characteristics	All population characteristics are described in Table S1 and include age (54-92 years old), height (1.53 -1.91 m), weight (59.60-124.80kg), BMI (21.31-40.29), type of primary treatment (5 active surveillance, 41 radical prostatectomy, 14 radiation, 8 De novo mets, 1 TULSA), medication history (17 prednisone, 2 dexamethasone, 11 metformin, 6 bicalutamide, 10 antibiotics), and presence of inflammatory bowel disease (4), pelvic radiation exposure (39) and metastatic disease (26). Patient treatment groups were as follows: No treatment (33), androgen deprivation therapy (21), androgen deprivation therapy combined with oral abiraterone acetate (14). These patient variables were included in multivariate analyses when assessing compositional differences in the microbiota (Table S2).
Recruitment	Patients were asked if they would be willing to donate a sample during routine checkup procedures. The 68 participants examined in our study were the only patients from the COSMIC cohort that were successfully recruited for purposes of microbiota analysis. Biases include the willingness to donate a fecal sample, which likely caused minimal impact on the study findings.
Ethics oversight	Research Ethics Board (REB) approval was obtained from our institution for this study under the Western University Research Project ID #108505. The REB is registered with the U.S. Department of Health & Human Services under the IRB registration number IRB 00000940.

Note that full information on the approval of the study protocol must also be provided in the manuscript.

Clinical data

Policy information about clinical studies

All manuscripts should comply with the ICMJEguidelines for publication of clinical research and a completedCONSORT checklist must be included with all submissions.

Clinical trial registration	NCT02364531
Study protocol	https://clinicaltrials.gov/ct2/show/NCT02364531
Data collection	All patient samples were collected at Victoria Hospital (London, Ontario, Canada) between 2017-2018.
Outcomes	This study focuses on the microbiota differences of patient groups and does not report on any of the primary or secondary outcomes of the COSMiC study (NCT02364531 trial).
